# A ten-year retrospective evaluation of acute flaccid myelitis at 5 pediatric centers in the United States, 2005–2014

**DOI:** 10.1371/journal.pone.0228671

**Published:** 2020-02-13

**Authors:** Margaret M. Cortese, Anita K. Kambhampati, Jennifer E. Schuster, Zaid Alhinai, Gary R. Nelson, Gloria J. Guzman Perez-Carrillo, Arastoo Vossough, Michael A. Smit, Robert C. McKinstry, Timothy Zinkus, Kevin R. Moore, Jeffrey M. Rogg, Meghan S. Candee, James J. Sejvar, Sarah E. Hopkins

**Affiliations:** 1 Division of Viral Diseases, National Center for Immunization and Respiratory Diseases, National Center for Emerging and Zoonotic Infectious Diseases, Centers for Disease Control and Prevention, Atlanta, Georgia, United States of America; 2 Contracting Agency to the Division of Viral Diseases, IHRC, Inc., Centers for Disease Control and Prevention, Atlanta, Georgia, United States of America; 3 Division of Infectious Diseases, Department of Pediatrics, Children’s Mercy Kansas City, Kansas City, Missouri, United States of America; 4 Division of Infectious Diseases, Department of Pediatrics, Alpert Medical School, Hasbro Children’s Hospital, Brown University, Providence, Rhode Island, United States of America; 5 Division of Child Neurology, Department of Pediatrics, Primary Children’s Hospital, University of Utah, Salt Lake City, Utah, United States of America; 6 Neuroradiology Section, Mallinckrodt Institute of Radiology, St. Louis Children’s Hospital, Washington University School of Medicine, St. Louis, Missouri, United States of America; 7 Department of Radiology, Children’s Hospital of Philadelphia, University of Pennsylvania, Philadelphia, Pennsylvania, United States of America; 8 Department of Radiology, Children’s Mercy Kansas City, Kansas City, Missouri, United States of America; 9 Department of Medical Imaging, Primary Children’s Hospital, University of Utah, Salt Lake City, Utah, United States of America; 10 Department of Diagnostic Imaging, Alpert Medical School, Hasbro Children’s Hospital, Brown University, Providence, Rhode Island, United States of America; 11 Division of High-Consequence Pathogens and Pathology, National Center for Emerging and Zoonotic Infectious Diseases, Centers for Disease Control and Prevention, Atlanta, Georgia, United States of America; 12 Division of Neurology, Children’s Hospital of Philadelphia, University of Pennsylvania, Philadelphia, Pennsylvania, United States of America; Universitatsspital Basel, SWITZERLAND

## Abstract

**Background:**

Acute flaccid myelitis (AFM) is a severe illness similar to paralytic poliomyelitis. It is unclear how frequently AFM occurred in U.S. children after poliovirus elimination. In 2014, an AFM cluster was identified in Colorado, prompting passive US surveillance that yielded 120 AFM cases of unconfirmed etiology. Subsequently, increased reports were received in 2016 and 2018. To help inform investigations on causality of the recent AFM outbreaks, our objective was to determine how frequently AFM had occurred before 2014, and if 2014 cases had different characteristics.

**Methods:**

We conducted a retrospective study covering 2005–2014 at 5 pediatric centers in 3 U.S. regions. Possible AFM cases aged ≤18 years were identified by searching discharge ICD-9 codes and spinal cord MRI reports (>37,000). Neuroradiologists assessed MR images, and medical charts were reviewed; possible cases were classified as AFM, not AFM, or indeterminate.

**Results:**

At 5 sites combined, 26 AFM cases were identified from 2005–2013 (average annual number, 3 [2.4 cases/100,000 pediatric hospitalizations]) and 18 from 2014 (12.6 cases/100,000 hospitalizations; Poisson exact p<0.0001). A cluster of 13 cases was identified in September–October 2014 (temporal scan p = 0.0001). No other temporal or seasonal trend was observed. Compared with cases from January 2005–July 2014 (n = 29), cases from August–December 2014 (n = 15) were younger (p = 0.002), more frequently had a preceding respiratory/febrile illness (p = 0.03), had only upper extremities involved (p = 0.008), and had upper extremity monoplegia (p = 0.03). The cases had higher WBC counts in cerebrospinal fluid (p = 0.013).

**Conclusion:**

Our data support emergence of AFM in 2014 in the United States, and those cases demonstrated distinctive features compared with preceding sporadic cases.

## Introduction

Acute flaccid myelitis (AFM) is a term developed in 2014 to describe a severe illness similar to paralytic poliomyelitis in clinical, electrodiagnostic, and radiological features, occurring primarily among children [1–4]. Clinical presentation includes acute flaccid limb weakness, sometimes with cranial nerve involvement or respiratory compromise. Passive AFM surveillance was established in the United States after a cluster of AFM was investigated in Colorado beginning August 2014 that occurred amidst an outbreak of severe respiratory disease caused by enterovirus-D68 (EV-D68) [2]. In 2014, an AFM case was defined as a person ≤21 years, with acute onset of limb weakness and with spinal cord magnetic resonance imaging (MRI) revealing lesions predominantly of the gray matter, and excluded those with spinal cord trauma or an otherwise known etiology of limb weakness [4]. Ultimately, 120 AFM cases were reported with onset during August–December 2014; direct laboratory evidence linking AFM with EV-D68 infection was inconclusive [4]. With the elimination of poliovirus in the United States (last outbreak in 1979)[5, 6] and the discontinuation of the live-attenuated oral polio vaccine (OPV)[6], notifications of poliomyelitis are rare. However, because there was no formal surveillance system in place to capture AFM cases prior to 2014, the extent to which 2014 was truly different from other years in the recent past was unknown and therefore formal retrospective evaluations were needed.

During the 2014 investigation it was noted that, in addition to the clinical similarities among the cases, the spinal cord MR imaging had consistent features, including longitudinal signal hyperintensities in the anterior spinal cord gray matter on T2-weighted images [7, 8]. Also, longitudinally extensive lesions that primarily involve the gray matter but may not be confined to the anterior horn cells, may be seen early in the course of AFM [7, 8]. These features were also critical in distinguishing AFM from other causes of acute flaccid paralysis. Hence, features of spinal MRIs could have high specificity for AFM and potentially be used as a surveillance tool for AFM to be followed by medical record review for clinical confirmation.

Continued U.S. surveillance under a standardized AFM case definition demonstrated much lower case numbers reported in 2015 and 2017, and increases in 2016 and 2018 with a seasonal peak of cases remarkably similar to 2014 [9–11]. To understand the occurrence and features of AFM in the recent past, which would inform investigations on causes of this potentially emerging condition, we performed a retrospective evaluation of AFM cases in different U.S. regions over a 10-year period.

## Materials and methods

We sought to identify 5 centers (number based on the funding available) that reported ≥1 AFM case during 2014, were from ≥3 different geographic regions, and were interested in and capable of performing a retrospective review. The 5 participating sites were from the Midwest (Children’s Mercy Kansas City, Kansas City, MO [CMKC]; St. Louis Children’s Hospital, St. Louis, MO [STL], Eastern US (Children’s Hospital of Philadelphia, Philadelphia, PA [CHOP]; Hasbro Children’s Hospital, Providence, RI [Hasbro]); and Western US (Primary Children’s Hospital, Salt Lake City, UT [PCH])(S1 Fig). Our goals were to first identify all possible AFM cases in patients aged ≤18 years based on the hospitals’ issued spinal cord MRI reports, and then review the MR images and medical charts to classify each as an AFM case consistent with 2014 CDC surveillance case definition [4], not an AFM case, or indeterminate. Search strategies were piloted and optimized at CHOP, involving review of 2,723 spinal MRI reports covering two years (S1 Appendix). Each hospital’s IRB (CMKC: Children’s Mercy IRB; STL: Washington University IRB; CHOP: Children’s Hospital of Philadelphia IRB; Hasbro: Lifespan IRB; PCH: University of Utah IRB and Intermountain Primary Children’s Hospital IRB) approved the protocol and waived the requirement for informed consent, including for data abstraction from medical charts. The medical charts were not anonymized at the sites before review by site investigators. The CDC Human Subjects Committee approved the protocol. Only anonymized data were provided to CDC from the sites.

Beginning in 2015, site investigators searched ICD-9 codes of electronic medical or billing records for January 1, 2005–December 31, 2014 to identify patients ≤18 years with inpatient or outpatient diagnosis of myelitis [323.0–323.99, 341.2–341.22], encephalomyelitis [323], encephalitis [323], meningitis [047.8, 047.9, 320.7, 320.0–322.90] or multiple sclerosis [340], and who had a spinal cord MRI report issued (S2 Fig). Additionally, a text search of all spinal MRI reports issued for persons ≤18 years during the 10-year period was conducted to identify scans ordered for any of 36 conditions included in the acute flaccid paralysis evaluation by Zangwill et al [12], or that contained those terms in the report (Table B in S1 Appendix). These subsets of MRI reports were read individually to identify those that documented any of four findings: myelitis, increased T2 signal in the spinal cord, cord edema, or cord infarction, without a known explanatory condition (e.g., tumor). If these criteria were met, corresponding MR images were reviewed by study neuroradiologists (at 1 site, experienced pediatric neurologist for some images) to determine if the spinal cord had area(s) of signal abnormality, and if yes, to identify those with largely grey matter involvement. They classified images as overall not consistent with AFM, consistent with AFM, or in the indeterminate category. For patients with images in the last two categories, brain MR images (if imaging performed), and medical charts were reviewed to establish if the clinical presentation was consistent with AFM (acute and flaccid paralysis, absence of upper motor neuron signs) without a clear alternate diagnosis (for instance, documented spinal cord trauma; brain MRI with white matter lesions and encephalopathy consistent with acute disseminated encephalomyelitis; neuromyelitis optica antibody positivity). Site investigators made a final case classification as AFM, not AFM, or indeterminate case, based on the imaging and clinical information.

Two procedural modifications occurred. At PCH, MRI reports from 2005–2008 could not be searched for the 36 terms, so possible cases for those years were detected by discharge ICD-9 codes as the first step. At STL, neuroradiologists were more inclusive and reviewed images from all patients identified by the search strategies.

We used annual hospitalizations in persons ≤18 years to reflect the catchment population size, and as denominators for describing AFM incidence rates (S1 Table). Temporal clustering was assessed using multivariate temporal scan statistic: 1-month windows, Monte Carlo replications to total 10,000 (SaTScan, version 9.4.4)[13]. Other analyses were performed using Stata 13 (StataCorp, College Station, TX).

## Results

At all sites combined, 48 patients had spinal MR images assessed as consistent with AFM and 43 had images in the indeterminate category (S1 Appendix). All had clinical records reviewed. Among the 48 patients with spinal MR images assessed as consistent with AFM, the final classification following clinical review was AFM in 38 (79%), indeterminate in 4 (8%), and not AFM in 6 (12%). Among the 43 patients with spinal MR images in the indeterminate category, the final classification was AFM in 8 (19%), indeterminate AFM in 9 (21%) and not AFM in 26 (60%). Of the resultant 46 with final classification of AFM, two were excluded from further analysis: one because weakness onset was in 2004, one because the patient did not receive neurological care at the site but had only a later MRI in 2007 and year of limb weakness onset was unknown. All cases met the CDC 2014 case definition[4].

**Fig 1:**
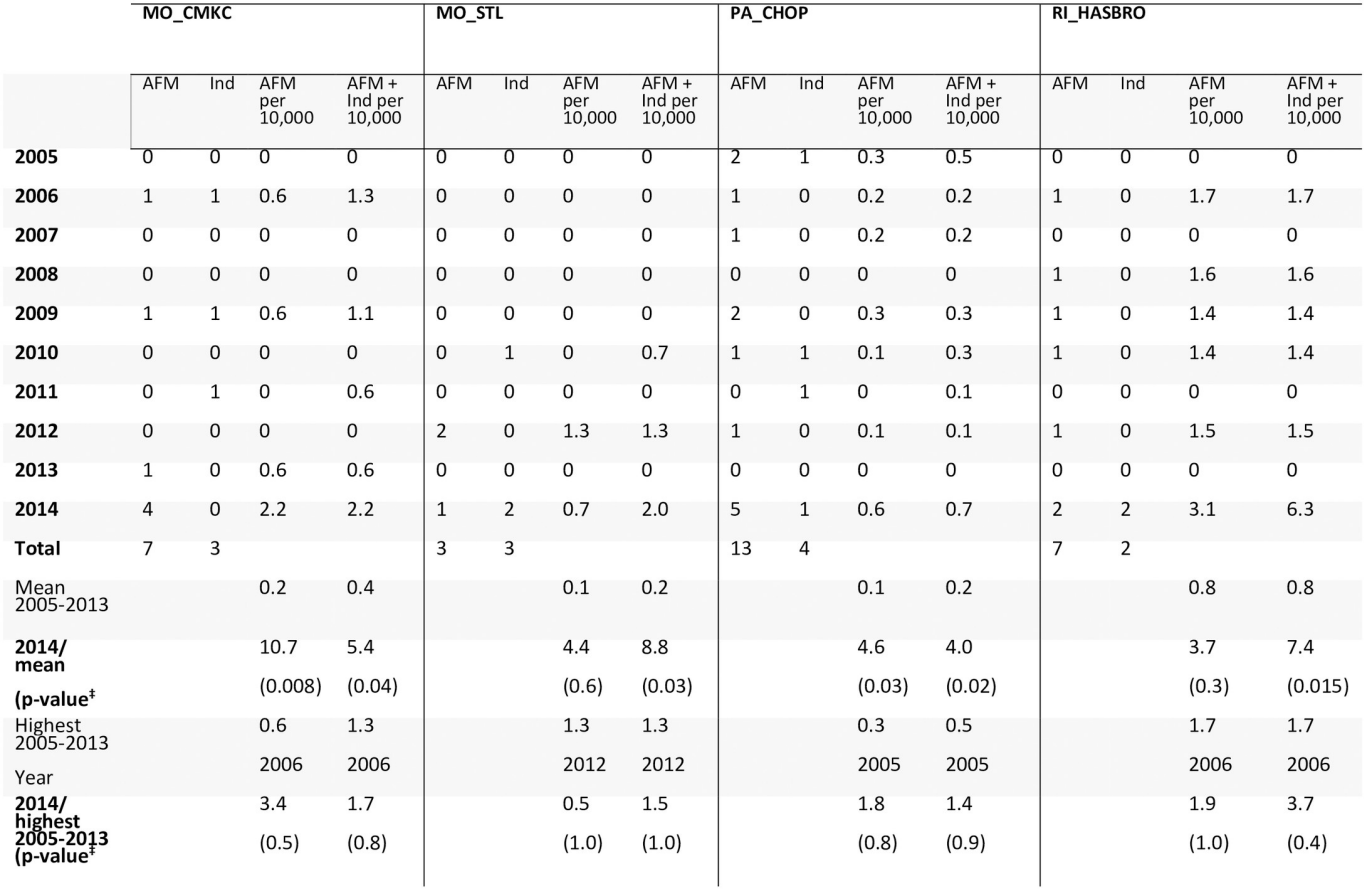
Number of AFM and indeterminate cases by calendar year and site among persons aged ≤18 years, and cases per 10,000 pediatric hospitalizations.

**Fig 2:**
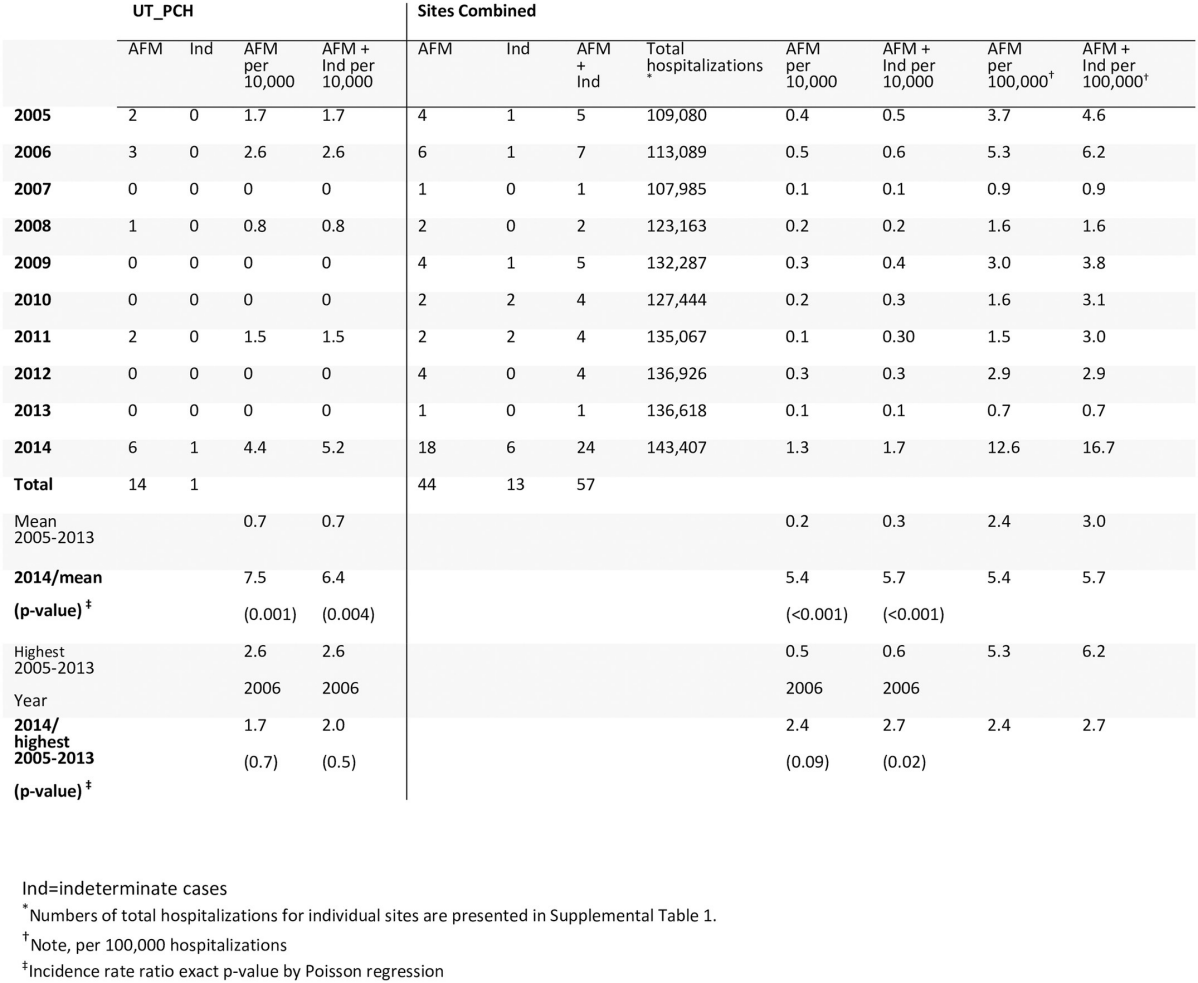
Number of AFM and indeterminate cases by calendar year and site among persons aged ≤18 years, and cases per 10,000 pediatric hospitalizations (continued).

**Fig 3:**
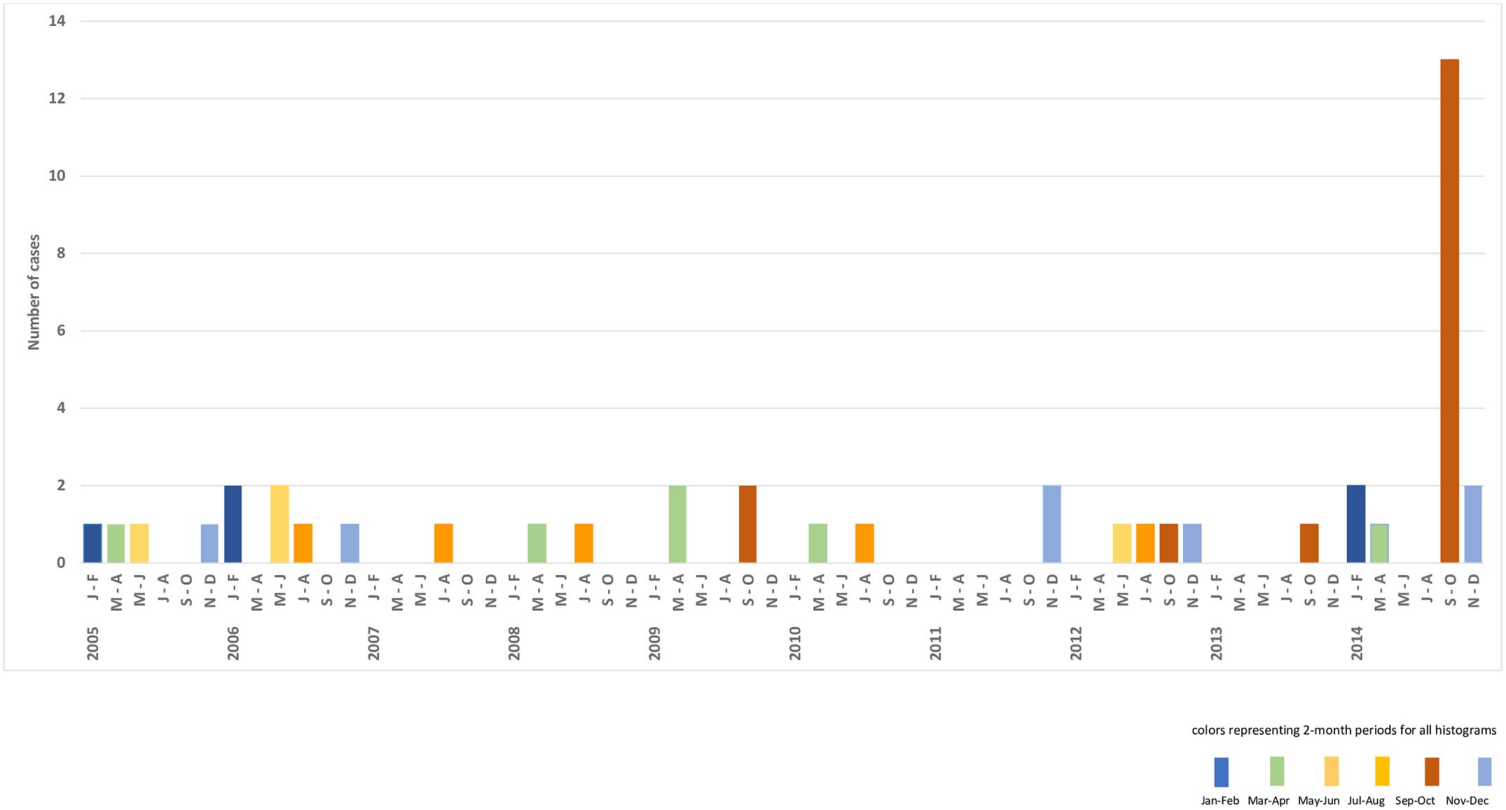
Number of AFM cases by 2-month periods, 5 sites combined.

Hence, 44 AFM cases were identified with onset during 2005–2014; individual sites had 3–14 cases (Figs 1–3). Twenty-six had onset during 2005–2013, with average annual number of 3 (range 1–6) and average annual rate of 2.4 cases per 100,000 hospitalizations (range 0.7–5.3). Eighteen had onset in 2014 (rate 12.6/100,000). The 2014 rate was 5.4 times the pre-2014 average annual rate (95% CI 2.8, 10.3; Poisson exact p<0.0001). The 2014 rate was 2.4 times that of the next highest year, 2006 (95% CI 0.90, 7.3; Poisson exact p = 0.09). Among sites individually, the 2014 rate was higher (3.7–10.7 times greater; Poisson exact p<0.05) than the pre-2014 annual average rate at 3 of the 5 sites (Figs 1 and 2).

**Fig 4:**
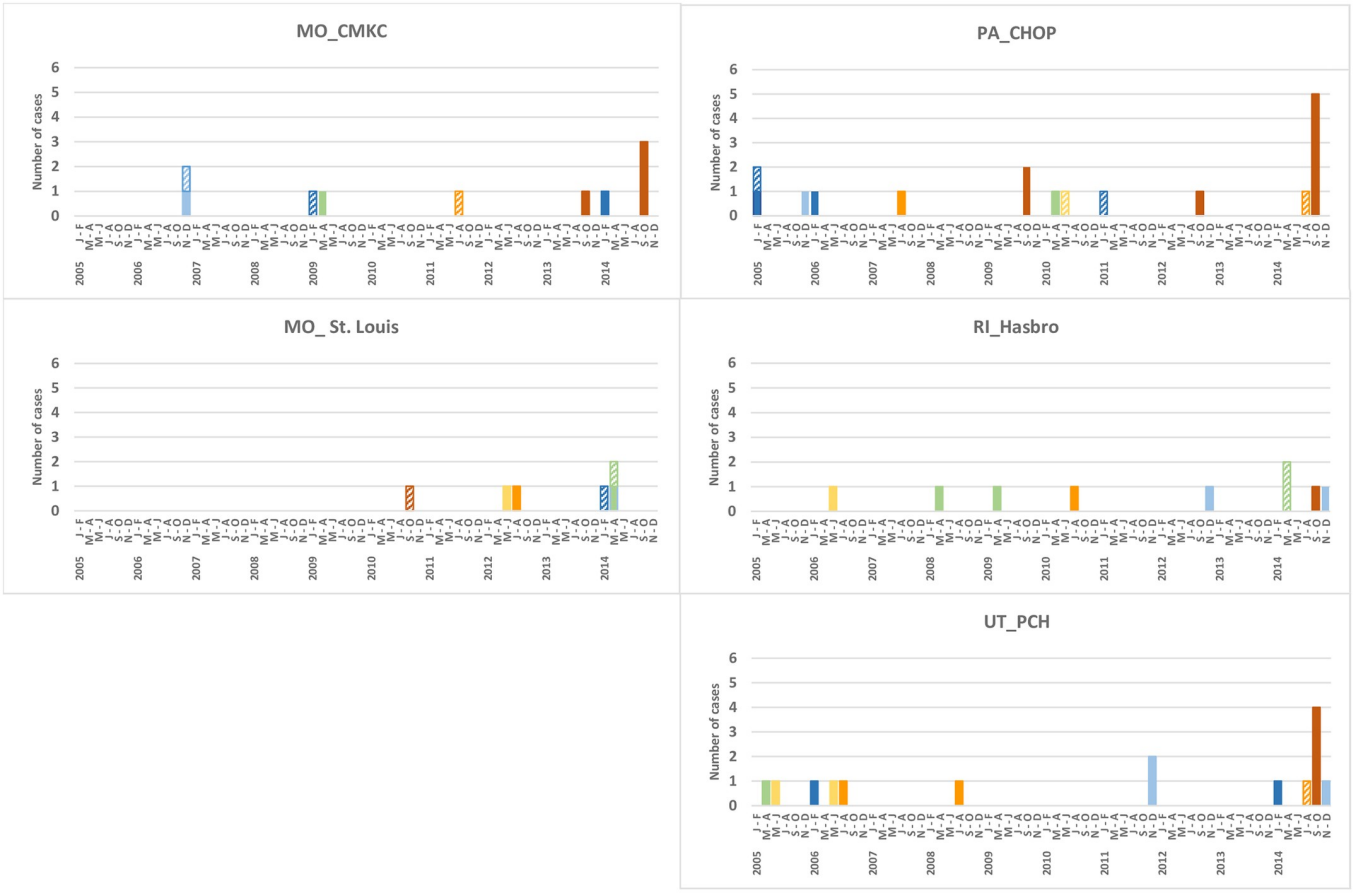
Number of AFM (solid columns) and indeterminate cases (hashed columns) by 2-month periods, by site. Color legend same as in Fig 3.

The majority (13/18, 72%) of 2014 cases occurred during September–October (Figs 3 and 4, S3 Fig) when 3 sites (CMKC, CHOP, PCH) had more cases than in any 2-month period of any previous year, and Hasbro had the same number (1 case). September–October 2014 was identified as the most likely cluster in the decade (expected cases versus observed cases = 0.8 versus 13; temporal scan p = 0.0001). Pre-2014, there was no periodicity or seasonal pattern evident for sites combined or individually (Fig 4, S3 Fig).

Seven indeterminate cases were identified during 2005–2013 and 6 were identified in 2014 (S4 Fig, S2 Table). At each site, the 2014 rate of ‘AFM cases plus indeterminate cases combined’ was higher than the site’s mean during 2005–2013 (Figs 1 and 2). Each site also had ≥2 years without an AFM or indeterminate case. The number of possible AFM cases with images reviewed and assessed as not AFM in 2014 was similar to mean annual number for 2005–2013 (S1 Appendix).

### Case characteristics

Because late 2014 appeared different and passive surveillance began with August 2014 cases, we distinguished two onset periods for analysis: January 2005–July 2014 (“period 1”, n = 29), and August–December 2014 (“period 2”, n = 15). Compared with period 1, period 2 cases were significantly younger (median 7.1 years vs 11.7 years, p = 0.002; Table 1, S5 Fig) and more likely to have a respiratory or febrile illness in the 2 or 4 weeks before limb weakness onset (4 weeks: 93% vs 58%, p = 0.03).

**Table 1:** Characteristics of AFM cases, by onset period.

Characteristic	Period 1: January 1, 2005–July 31, 2014	Period 2: August 1, 2014–December 31, 2014	p-value
	N = 29	N = 15	
Age, median (range)	11.7 years (1.2–17.9)	7.1 years (0.4–14.2)	**.002**
Sex	Male: 14 (48%)	Male: 11 (73%)	.20
	Female: 15 (52%)	Female: 4 (27%)	
Race*	White 24/28 (86%)	White 10/11 (91%)	1.0
	Black 3/28 (11%)	Black 1/11 (9%)	
Underlying medical condition	4/28 (14%)^†^	3/15 (20%)^†^	.68
Underlying asthma	1/29 (3%)^†^	3/15 (20%)^†^	.11
Received steroids in the 4-weeks before weakness onset	1/29 (3%)^‡^	2/13 (15%)^‡^	.22
Autoimmune disorders in immediate family members	2/26 (8%)^§^	1/13 (8%)^§^	1.0
Illness in the 4-weeks preceding limb weakness onset:			
Respiratory illness	10/25 (40%)	12/15 (80%)	**.02**
Febrile illness	7/24 (29%)	7/13 (54%)	.17
Respiratory or febrile illness	15/26 (58%)	14/15 (93%)	**.03**
GI illness	6/27 (22%)	2/14 (14%)	.69
Illness in the 2-weeks preceding limb weakness onset			
Respiratory illness	7/23 (30%)	9/13 (69%)	**.04**
Febrile illness	6/23 (26%)	7/13 (54%)	.15
Respiratory or febrile illness	11/23 (47%)	11/13 (85%)	**.04**
Fever on day of weakness onset	2/23 (9%)	2/11 (18%)	.58
**Clinical involvement**	n = 29^‖^	n = 15^¶^	
Upper extremity(ies) involved	19/29 (66%)	12/14 (86%)	.29
Lower extremity(ies) involved	25/28 (89%)	8/15 (53%)	.**02**
Only upper extremity(ies) involved	3/28 (11%)	7/14 (50%)	**.008**
Monoplegia/paresis	2/28 (7%)	5/14 (36%)	.**03**
Only lower extremity(ies) involved	11/29 (38%)	2/13 (15%)	.28
Monoplegia/paresis	1/29 (3%)	0/13 (0%)	1.0
Upper and lower extremities involved, but not all 4	3/28 (11%)	2/14 (14%)	1.0
Quadriplegia/paresis	11/28 (39%)	3/15 (20%)	.31
Any cranial nerve sign**	7/27 (26%)	3/15 (20%)	1.0
Required assisted ventilation (including BiPAP, CPAP)	6/29 (21%)	2/15 (13%)	.70
**CSF characteristics**	n = 25 (25/29, 86%)	n = 14 (14/15, 93%)	
WBC count, median (range)	4 cells/μL (0–360)	33 cells/μL (1–134)	**.013**
WBC >5 cells/uL	12/25 (48%)	12/14 (86%)	.**04**
Lymphocytic predominance^††^	7/12 (58%)	7/11 (64%)	1.0
WBC >5 cells/uL at ≤2 days^‡‡^	6/17 (35%)	7/8 (88%)	.**03**
Protein, median (range)	31 mg/dL (12–95)	43 mg/dL (20–128)	.05
Protein >45 mg/dl	7/25 (28%)	5/14 (36%)	.72
Glucose, median (range)	61 mg/dL (44–113)	56 mg/dL (47–84)	.43

Denominators are number of cases with information. 2-sided Fisher’s exact p-value or p-value by Kolmogorov-Smnirov test presented.

P-values not adjusted for multiple comparisons.

*1 case from period 1 is of other race. Information on Hispanic ethnicity: 1/15 (6%) period 1 (also indicated as white race); 4/11 (36%) period 2, 1 of whom also indicated as white race.

^†^Period 1: 1 patient each with asthma, 1 with acute tubular interstitial nephritis, 1 with cocaine exposure in utero and developmental delay; 1 with mitral valve regurgitation and seasonal allergies (not counted: 1 with only seasonal allergies) Period 2: 2 patients with asthma alone, one with asthma and obesity (not counted: 1 with eczema, 1 with attention deficit disorder

^‡^Period 1: 1 patient admitted in status asthmaticus, intubated and treated with parental steroids was found to have limb weakness after extubtation. Period 2: 1 patient was receiving parenteral steroids when limb weakness developed; 1 patient received oral steroids

^§^Period 1: mothers of 2 patients had lupus, one of whom also had Crohn’s; Period 2: brother of one patient had acute necrotizing encephalopathy of childhood.

^‖^Information on lower extremity involvement was unknown for 1 patient

^¶^For 2 patients, information on involvement of 1 limb was not available.

**Period 1 (exclusive categories): dysarthria, n = 2; dysarthria and facial weakness, n = 1; dysphagia, n = 1; facial weakness, n = 2; diploplia, n = 1; Period 2: dysphagia, dysarthria and facial weakness, n = 1; neck and ocular muscle weakness = 1; neck weakness, n = 1.

^††^Lymphocytes>50%, of those with WBC>5 cells/uL and differential provided.

^‡‡^Restricted to patients with lumbar puncture performed 2 days or less after limb weakness onset. CSF RBC counts all <30 cells/uL in period 2

Among all cases, 72% (31/43) had at least upper extremity paresis/paralysis and 33% (14/43) had involvement of all limbs. The pattern of limb involvement differed by period—compared with cases from period 1, cases from period 2 were more likely to have weakness restricted to upper extremities (50%[7/14] vs 11% [3/28], p = 0.008) and have upper extremity monoplegia (36% [5/14] vs 7% [2/28], p = 0.03). The proportion with cranial nerve involvement (period 2: 20% [3/15]; period 1: 26% [7/27]) or who required assisted ventilation (period 2: 13% [2/15]; period 1: 21% [6/29]) were similar in both periods (Table 1).

There were no notable differences in spinal cord involvement as assessed by MRI by period (S3 Table). Cervical cord was most commonly involved (period 2: 86% [12/14]; period 1: 83% [20/24], with restriction to cervical cord in 40% [6/15] of cases from period 2 and 38% [11/29] from period 1. Among all patients, 83% (34/41) had lesions involving ≥3 spinal segments. Among patients who had brain MR imaging with gadolinium administered, no lesions were found in 61% (8/13) of cases from period 2 and 58% (14/24) of those from period 1. Among those with abnormal brain imaging, findings were similar for cases in periods 2 and 1, and infratentorial lesions were most common (S3 Table).

Cases in period 1 had lower CSF WBC counts (median, 4 cells/uL) than cases in period 2 (median, 33 cells; p-value 0.013; CSF was collected a median number of 2 days after weakness onset for each period (Table 1, S6 Fig); of cases with CSF obtained ≤2 days after weakness onset, only 35% (6/17) of cases from period 1 had >5 cells/uL, compared with 88% (7/8) of cases from period 2 (p = 0.03). No AFM case had a pathogen identified in CSF, according to medical records. The patients’ clinicians considered the responsible pathogen to likely be *Mycoplasma* in 2 cases (one 2006 case, based on positivity of serum IgM; one 2008 case, based on positivity of serum IgM and increase in serum IgG titer), and cytomegalovirus in one (2008 case, based on positivity of serum IgM and IgG). For the remainder, clinicians had not concluded they had identified a responsible pathogen.

## Discussion

At our 5 referral centers combined, we found AFM cases had occurred each year, with low and seemingly sporadic cases prior to 2014, and our data support a higher occurrence in 2014. Strengths of our study include representation from 3 U.S. areas, 10-year timeline, and comprehensive search strategies including >37,000 spinal MRIs. Given the rarity of cases, changes in case counts by even a few in a year can appear substantial. However, by ascertaining case timing during the 9.7 years before August 2014, the clustering in September–October 2014 is striking and unlike that in any other period during 2005–2013. This clustering was observed across the 3 areas.

Two other U.S. locations have reported longitudinal AFM data including 2014. Data from Children’s Hospital, Colorado (July 1, 2010–October 31, 2014; pre-August 2014 cases identified by discharge ICD-9 codes, followed by MR image review)[2] and California (June 1, 2012–July 31, 2015, voluntary, passive surveillance by California Department of Public Health [3]) demonstrated cases in each earlier year and clustering in fall 2014. Combined—our evaluation, Colorado and California reports, and the US surveillance (to which 17 other states had reported ≥2 cases during August–December 2014 [4])−the available data support that fall 2014 was unusual for AFM in the United States. Since the cluster first reported by Colorado, CDC has supported AFM surveillance through state health departments.

This timing suggests that the cases in the fall of 2014 resulted from an exposure that was geographically wide and occurred during a tight time-window. An infectious trigger is supported by the high frequency of preceding respiratory/febrile illness and the CSF pleocytosis, and the younger age suggests possible age cohorts without previous exposure. At the STL site, no case was identified from fall 2014. This may be a true finding and a consequence of expected variability in circulation of the causative pathogen(s) at the local level. It is possible, however, that there may have been an unidentified error in procedures. The overlap in timing of 2014 AFM cases with EV-D68 respiratory disease in the United States [4, 14–16], data from Colorado cases vs control groups [17], and similar epidemiological picture from Japan during fall 2015 [18], suggest that these AFM clusters were associated with EV-D68, but data were not conclusive. Subsequently, the highest number of cases reported to the US surveillance system occurred in late summer/fall 2018 [9] temporally associated with EV-D68 detection in respiratory specimens [19]. However, among the subset of 74 cases from 2018 with CSF specimens tested at CDC, a pathogen was identified in only 2 cases (EV-D68 and EV-A71 in one CSF specimen each) [20, 21]. Non-poliovirus AFM cases have also been reported from several other countries in the recent past [22–26]. The clinical and radiological phenotype termed AFM can be caused by pathogens other than polioviruses, including other enteroviruses (EV-A71) and certain flaviviruses (West Nile virus) [27–29]. In our retrospective series, there was no obvious seasonality before 2014 that would suggest particular pathogens. However, our study identified 2 AFM cases at CHOP in September 2009. These cases coincided with the institution’s increase in children with respiratory disease whose respiratory specimens yielded enterovirus; EV-D68 was detected in 42% of 66 specimens characterized at CDC [30]. Before 2014, September 2009 was the only month when CHOP had >1 AFM case.

Similarities and differences between our period 1 and period 2 cases may provide insights into the pathogen(s) and disease processes that result in AFM. In both periods, upper extremities were most affected, different from paralytic poliovirus disease in which most patients have lower extremity involvement [31–33]. The occurrence of monoplegia/monoparesis (period 1: upper extremity in 2 patients, lower extremity in 1 patient; period 2: upper extremity in 5 patients) is more characteristic of focal CNS involvement, such as that seen with neurotropic infectious pathogens rather than a post-infectious immune-mediated process. Monoplegia has been reported in some AFM cases from most case series covering 2014 and later, most commonly an upper extremity, reflecting the common cervical cord involvement [17, 18, 22–26]. The CSF WBC counts were low in many period 1 cases, which may suggest differences in pathogenesis between period 1 and period 2 cases. CSF pleocytosis is not common at onset in spinal infarct, which can appear identical to AFM on MRI, but is unlikely to have been a predominant cause of period 1 cases given the rarity of cord infarction in this age group [1, 34, 35].

Our study has limitations. First, site investigators could not be blinded to case time-period. Case classification involved judgement of radiological and clinical information and so it is plausible that possible 2014 cases were more likely to be designated cases rather than non-cases or indeterminate. There were few indeterminate cases, however, and including these yields the same conclusions regarding 2014. Also reassuring is that the annual number of possible cases assessed as not AFM was similar in 2014 versus earlier years. Second, during recruitment, we only contacted hospitals familiar with AFM, demonstrated by their reporting of possible cases to CDC in 2014 and hence our sites may not be representative of all US children’s centers. However, some other hospitals contacted that did not participate had reported greater case-numbers in 2014 than our sites, so it is unlikely that our 2014 findings were unique to our set of reporting hospitals. Third, At PCH, only ICD-9 code searches were performed for 2005–2008. Both search strategies were used for final 6 years, however, and conclusions are the same based on that period. Fourth, there may have been differences across sites particularly with the categorization of spinal MR images only (i.e., without any clinical information) to indeterminate category, and final classifications as indeterminate AFM cases.

Globally collaborative research and surveillance, with consensus inclusion and exclusion criteria for consistent reporting, will be important for investigating this emerging, severe illness.

## Supporting information

S1 Fig Location of participating hospitals. (TIF)

S2 Fig Procedures to identify AFM cases. (TIF)

S3 Fig Depiction of seasonality: AFM (solid columns) and indeterminate (hashed columns) cases by calendar month, 2005–2013 combined and 2014. (TIF)

S4 Fig Number of AFM (solid columns) and indeterminate (hashed columns) cases by 2-month periods, 5 sites combined. (TIF)

S5 Fig Age distribution and gender of AFM cases, by period. (TIF)

S6 Fig Cerebrospinal fluid WBC counts of AFM cases by time from limb weakness onset, by period. (TIF)

S1 Table Total number of hospitalizations among persons aged ≤18 years, by site and year. (DOCX)

S2 Table Characteristics of indeterminate cases. (DOCX)

S3 Table Characteristics of spinal and brain MRIs among AFM cases, by period. (DOCX)

S1 Appendix Pilot study and additional methods. (DOCX)
