# Supplementary figures and images for: A ten-year retrospective evaluation of acute flaccid myelitis at 5 pediatric centers in the United States, 2005–2014
Source: PLoS One. 2020 Feb 13;15(2):e0228671. doi: 10.1371/journal.pone.0228671 (PMC7018000; doi:10.1371/journal.pone.0228671)

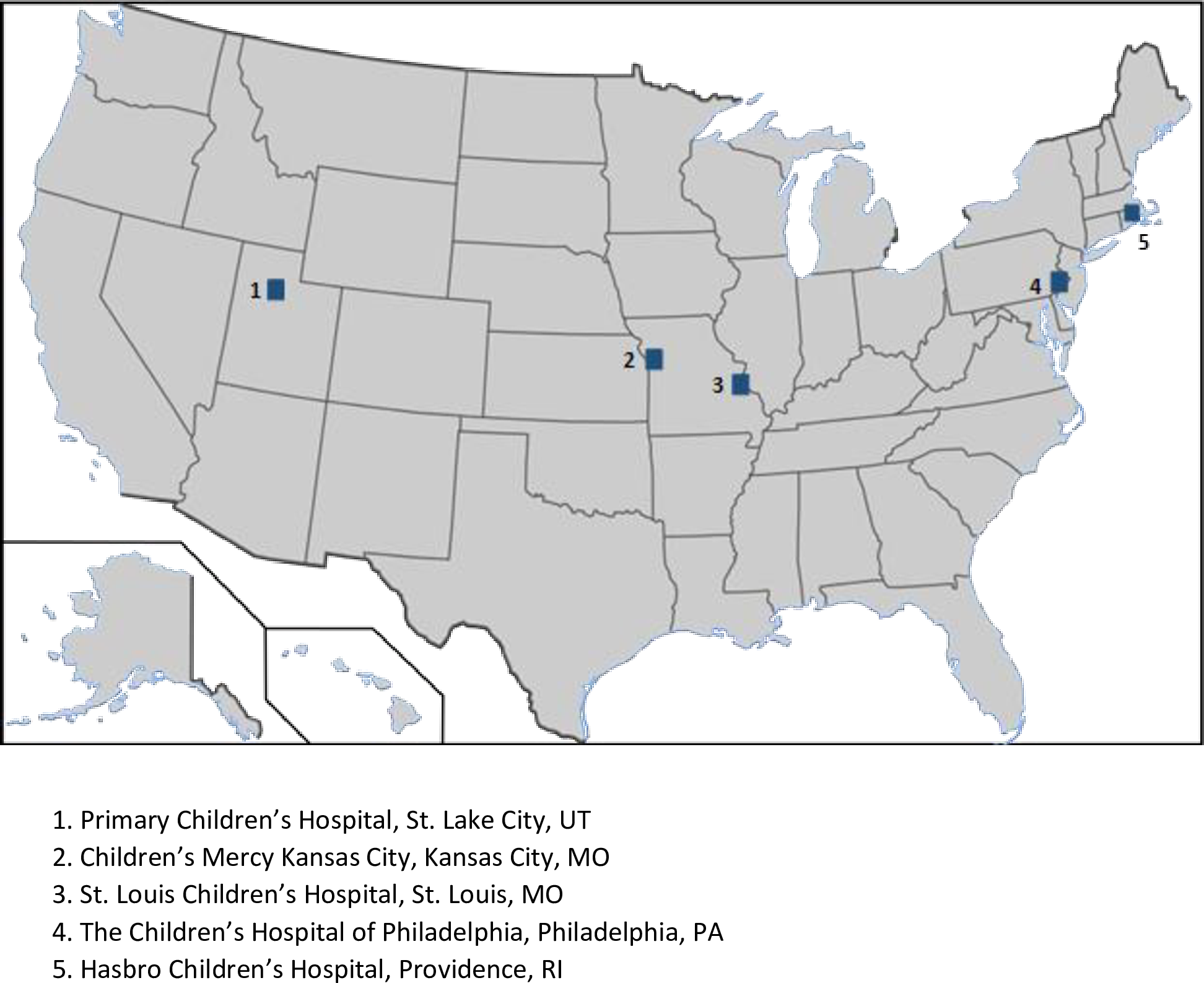

Supplement: S1 Fig — (TIF) [file pone.0228671.s001.tif]

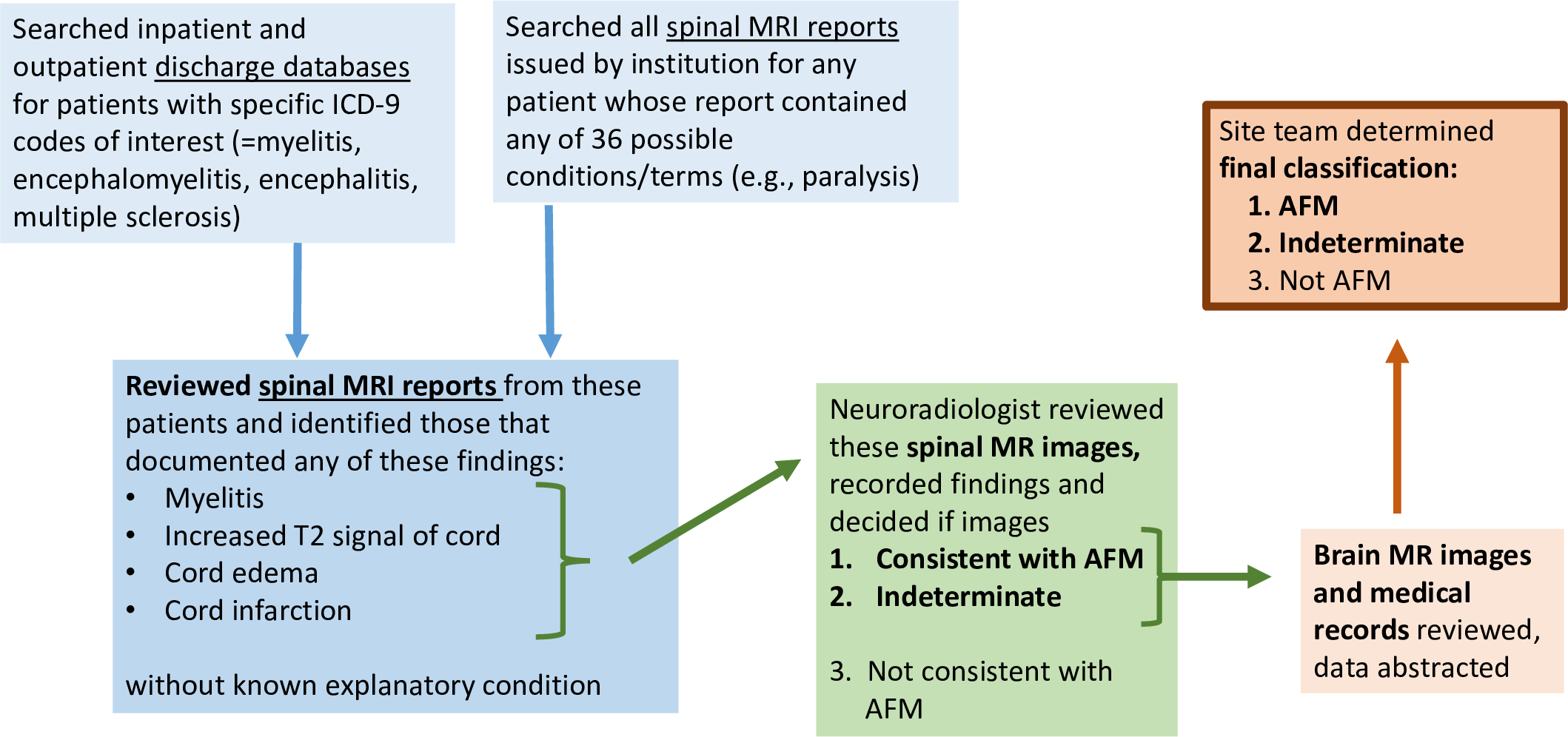

Supplement: S2 Fig — (TIF) [file pone.0228671.s002.tif]

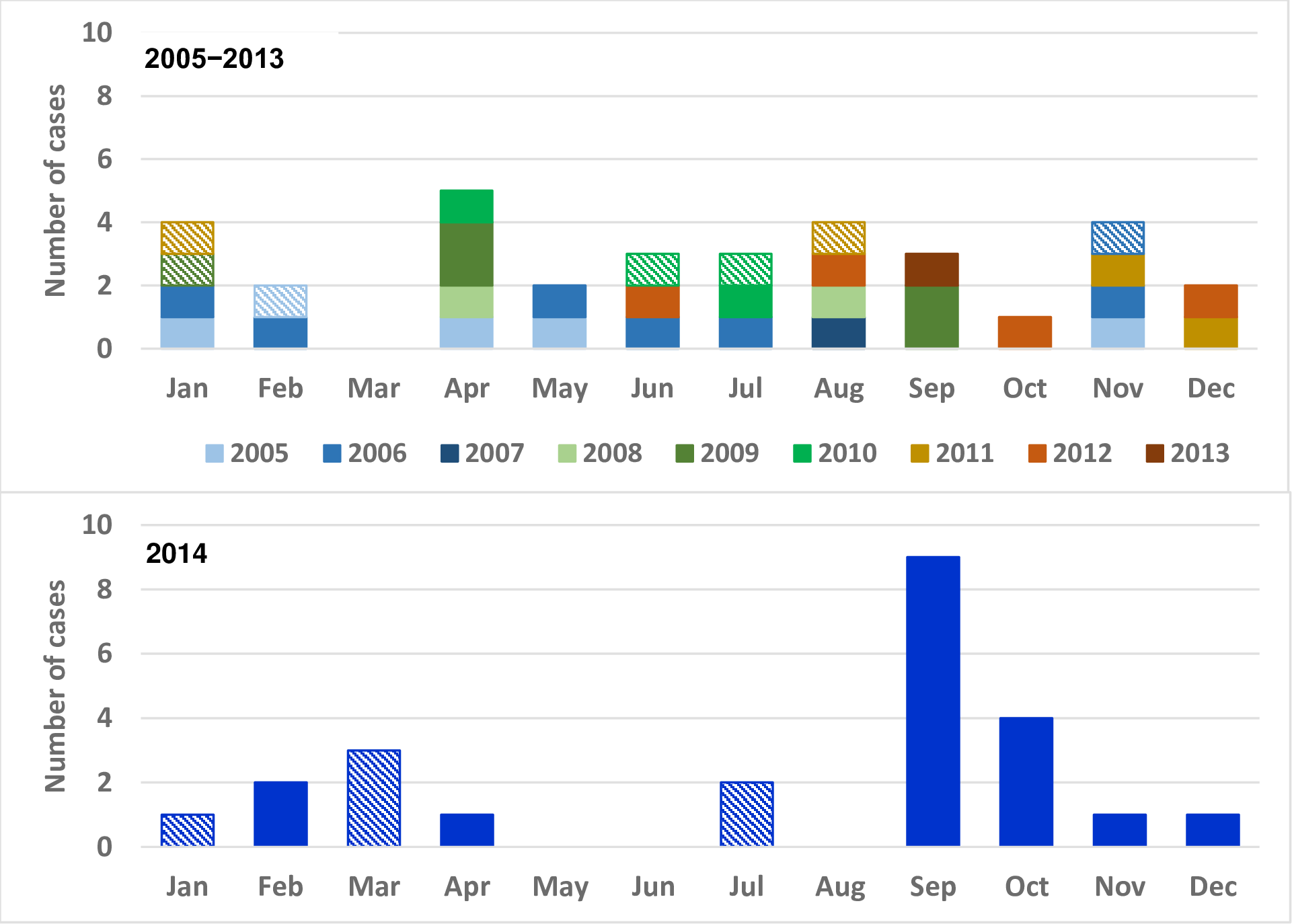

Supplement: S3 Fig — (TIF) [file pone.0228671.s003.tif]

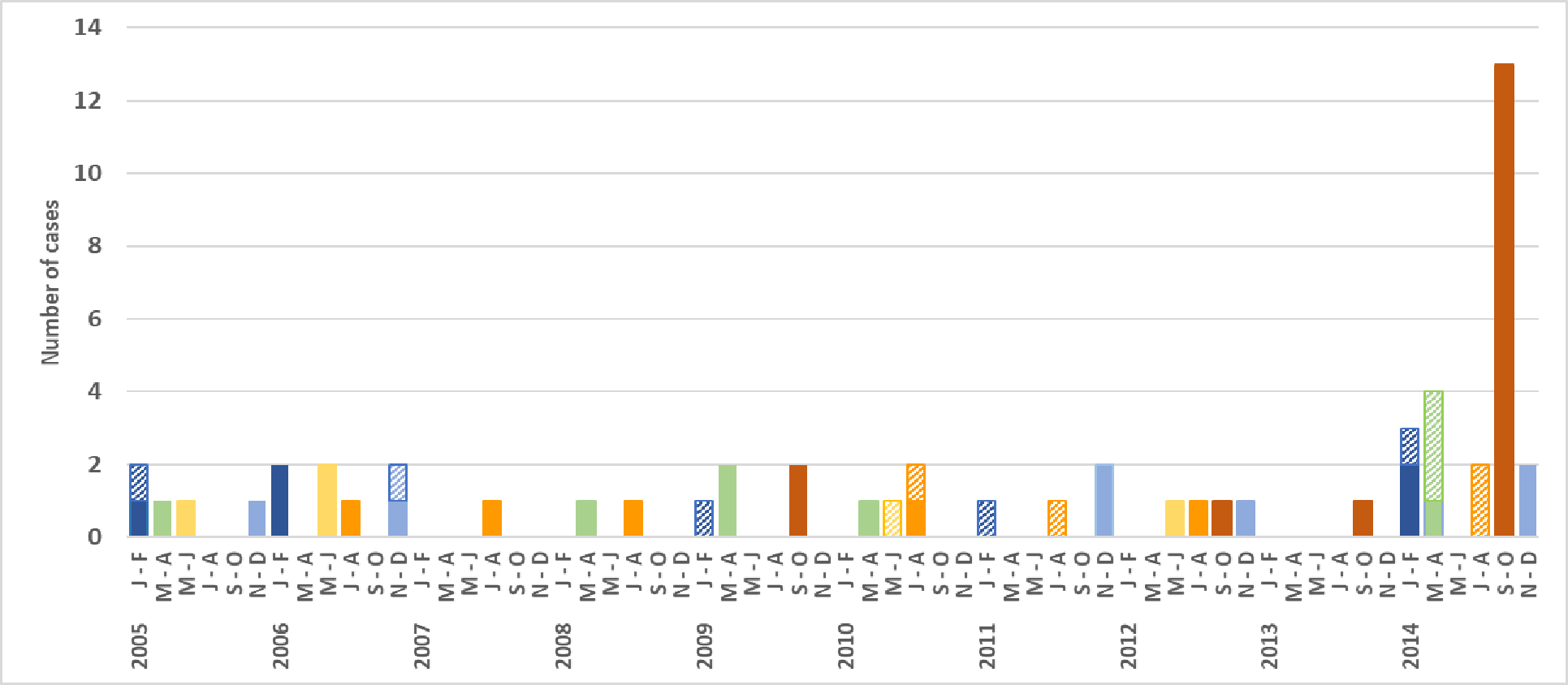

Supplement: S4 Fig — (TIF) [file pone.0228671.s004.tif]

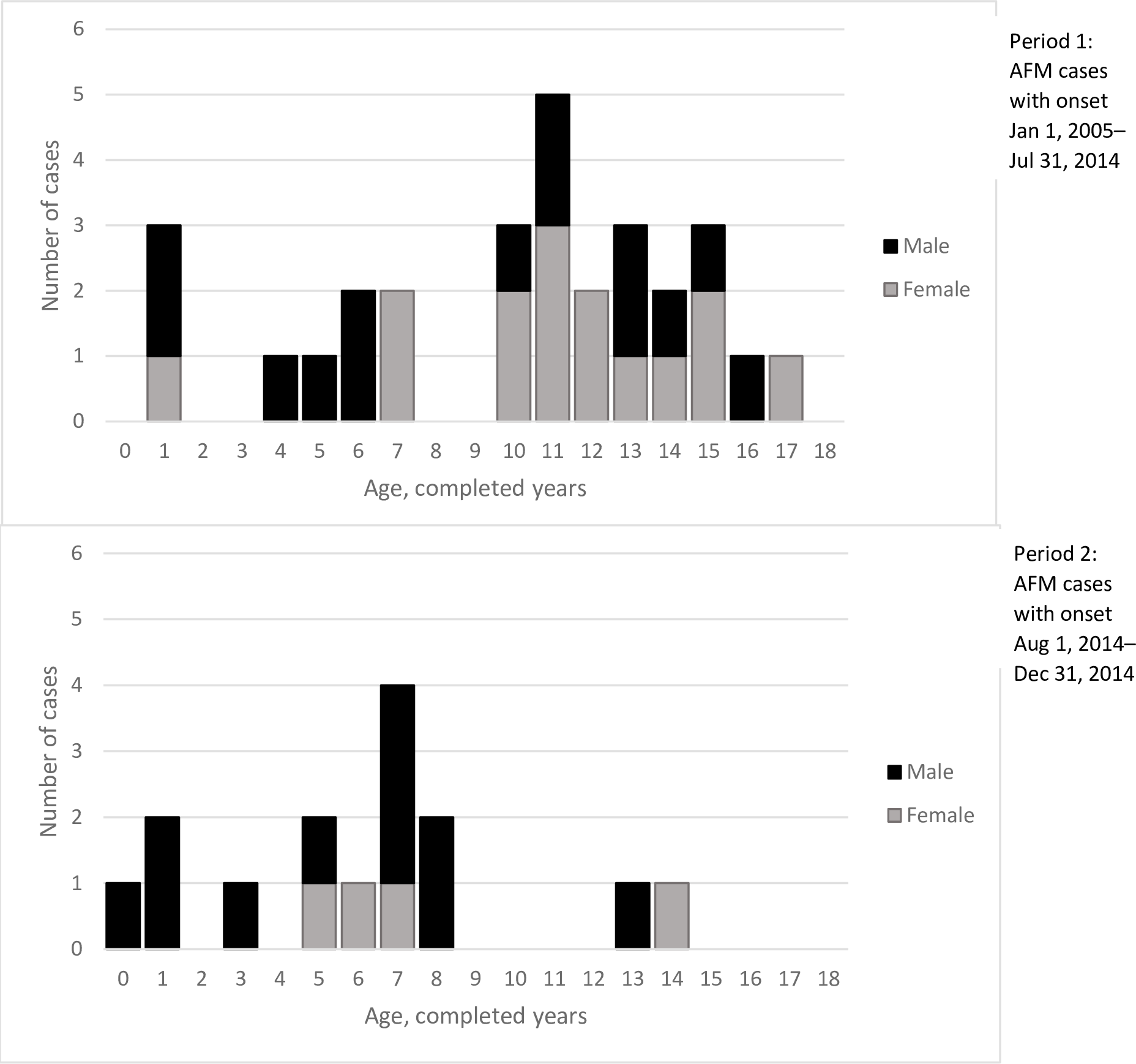

Supplement: S5 Fig — (TIF) [file pone.0228671.s005.tif]

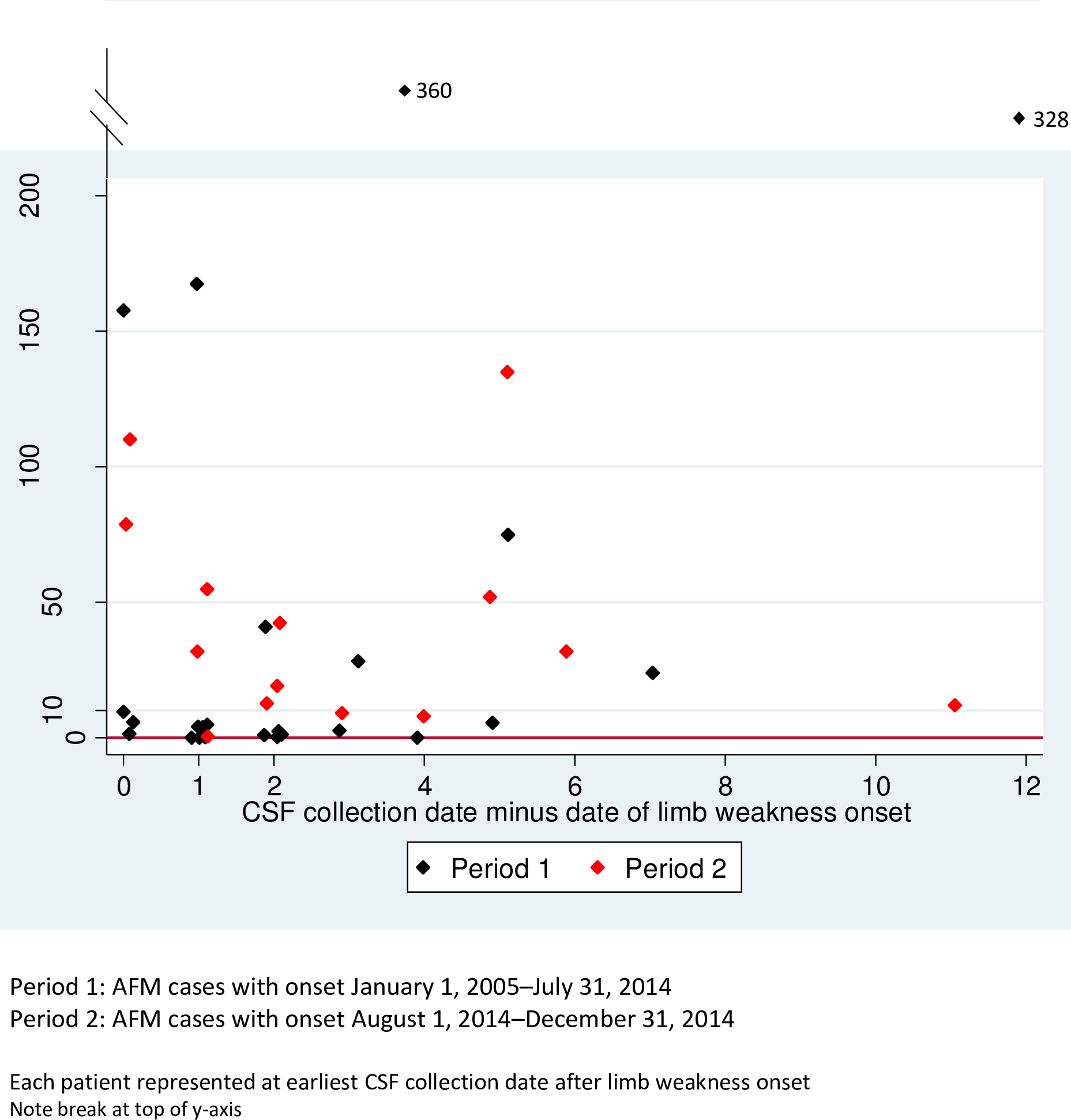

Supplement: S6 Fig — (TIF) [file pone.0228671.s006.tif]
