# Supplementary material for: A ten-year retrospective evaluation of acute flaccid myelitis at 5 pediatric centers in the United States, 2005–2014
Source: PLoS One. 2020 Feb 13;15(2):e0228671. doi: 10.1371/journal.pone.0228671 (PMC7018000; doi:10.1371/journal.pone.0228671)
Supplement: S1 Table — (DOCX) [file pone.0228671.s007.docx]

|  | MO_CMKC | MO_STL | PA_CHOP^*^ | RI_Hasbro | UT_PCH |
| --- | --- | --- | --- | --- | --- |
| 2005 | 12,411 | 14,190 | 64,595 | 6,205 | 11,679 |
| 2006 | 15,593 | 14,478 | 65,394 | 5,922 | 11,702 |
| 2007 | 16,544 | 13,999 | 58,629 | 6,208 | 12,605 |
| 2008 | 17,366 | 14,846 | 71,965 | 6,217 | 12,769 |
| 2009 | 17,399 | 15,116 | 79,567 | 6,970 | 13,235 |
| 2010 | 16,875 | 15,194 | 75,452 | 6,959 | 12,964 |
| 2011 | 17,075 | 14,932 | 82,781 | 7,349 | 12,930 |
| 2012 | 17,164 | 15,218 | 83,620 | 6,879 | 14,045 |
| 2013 | 17,143 | 15,219 | 84,480 | 6,295 | 13,481 |
| 2014 | 18,444 | 15,174 | 89,885 | 6,380 | 13,524 |

^*^Hospitalizations from 2005—2007 may be undercounted--administrative data systems were modified after this period.
