# Supplementary material for: A ten-year retrospective evaluation of acute flaccid myelitis at 5 pediatric centers in the United States, 2005–2014
Source: PLoS One. 2020 Feb 13;15(2):e0228671. doi: 10.1371/journal.pone.0228671 (PMC7018000; doi:10.1371/journal.pone.0228671)
Supplement: S2 Table — (DOCX) [file pone.0228671.s008.docx]

| **Characteristic** | **Onset January 1, 2005–**  **July 31, 2014; N=13** |
| --- | --- |
| Age, median (range) | 11.9 years (0.8-16.3) |
| Sex | Male: 7 (53%) Female: 6 (46%) |
| Race | White 13/13 (100%)^†^ |
| Underlying medical condition | 1/12 (8%)^‡^ |
| Underlying asthma | 0/12 (0%) |
| Received steroids in the 4-weeks before weakness onset | 0/9 (0%) |
| Autoimmune disorders in immediate family members | 2/9 (22%)^§^ |
| Illness in the 4-weeks preceding limb weakness onset: |  |
| Respiratory illness | 2/13 (15%) |
| Febrile illness | 1/10 (10%) |
| Respiratory or febrile illness | 3/12 (25%) |
| GI illness | 3/13 (23%) |
| Fever on day of weakness onset | 3/12 (25%) |
| **Clinical involvement** |  |
| Upper extremity(ies) involved | 4/13 (31%) |
| Lower extremity(ies) involved | 13/13 (100%) |
| Only upper extremity(ies) involved | 0/13 (0%) |
| Only lower extremity(Ies) involved | 9/13 (69%) |
| Monoplegia/paresis | 2/13 (15%) |
| Upper and lower extremities, but not all 4 | 1/13 (8%) |
| Quadriplegia/paresis | 3/13 (23%) |
| Any cranial nerve sign | 1/13 (8%) |
| Required assisted ventilation | 1/13 (8%) |
| **CSF characteristics** | n=11 (11/13, 85%)^‖^ |
| WBC count, median (range) | 6 cells/µL (0-180) |
| WBC >5 cells/uL | 6/11 (55%) |
| Lymphocytic predominance | 5/6 (83%) |
| WBC >5 cells/uL at ≤2 days | 2/8 (25%) |
| Protein, median (range) | 42 mg/dL (23-66) |
| Protein >45 mg/dl | 5/10 (50%) |
| Glucose, median (range) | 62 mg/dL ((47-148) |

Denominators are number of cases with information. Age collected as completed years and months.

All indeterminate cases were in the first time period, onset January 1, 2005-July 31, 2014

^†^Hispanic ethnicity: 0/10 with information provided. ^‡^1 patient with past history of chronic Bell’s palsy, urinary retention and fecal incontinence. Not counted: 1 patient each: Raynaud’s syndrome, allergic rhinitis, seasonal allergies ^§^ 1 father with psoriasis and arthritis; 1 father with inflammatory bowel disease ^‖^Excludes results for one patient with CSF collection 94 days after weakness onset
