# Supplementary material for: A ten-year retrospective evaluation of acute flaccid myelitis at 5 pediatric centers in the United States, 2005–2014
Source: PLoS One. 2020 Feb 13;15(2):e0228671. doi: 10.1371/journal.pone.0228671 (PMC7018000; doi:10.1371/journal.pone.0228671)
Supplement: S3 Table — (DOCX) [file pone.0228671.s009.docx]

| **Characteristic** | **Period 1:**  **January 1, 2005 – July 31, 2014** | | **Period 2:**  **August 1, 2014 – December 31, 2014** |
| --- | --- | --- | --- |
|  | **N=29** | | **N=15** |
| Spinal cord MRI | |  |  |
| Location of spinal cord involvement^*^ | |  |  |
| Cervical cord | | 20/24 (83%) | 12/14 (86%) |
| Thoracic cord | | 15/27 (56%) | 9/13 (69%) |
| Conus | | 8/26 (31%) | 4/12 (33%) |
| Cervical cord only | | 11/29 (38%) | 6/15 (40%) |
| Thoracic cord only | | 3/29 (10%) | 2/15 (13%) |
| Conus only | | 3/29 (12%) | 0/15 (0%) |
| Cervical, thoracic and conus | | 2/29 (7%) | 3/15 (20%) |
| Cervical and thoracic only | | 7/29 (24%) | 3/15 (20%) |
| Thoracic and conus only | | 3/29 (10%) | 1/15 (6%) |
| More than 1 discrete lesion^†^ | | 5/29 (17%) | 1/15 (13%) |
| Any spinal cord abnormality ≥3 segments in length^‡^ | | 20/26 (77%) | 14/15 (93%) |
| Maximal involvement on axial images^§^ | |  |  |
| Limited to anterior horn cells^‖^ | | 10/28 (36%) | 4/15 (27%) |
| All central gray +/- some white matter | | 16/28 (57%) | 11/15 (73%) |
| Any spinal nerve root enhancement | | 3/28 (11%)^¶^ | 3/15 (20%)^¶^ |
| Any spinal lesion with GAD enhancement | | 6/29 (21%) | 3/15 (20%) |
| Brain MRI | |  |  |
| No brain MRI within 30 days of first spinal MRI | | 2/29 (7%) | 2/15 (13%) |
| Had brain MRI within 30 days and imaged with GAD | | 24/29 (83%) | 13/15 (87%) |
| No T2 hyperintensity | | 14/24 (58%) | 8/13 (61%) |
| T2 hyperintensity in: | |  |  |
| only brainstem | | 1/24 (4%) | 2/13 (15%) |
| only brainstem and contiguous upper cord | | 3/24 (12%) | 2/13 (15%) |
| central deep gray nuclei | | 4/24(17%) | 0/13 (0%) |
| supratentorial white matter | | 2/24 (8%) | 1/13 (8%) |
| cerebellum | | 3/24(12%) | 1/13 (8%) |
| Had brain MRI within 30 days and imaged without GAD | | 3/29 (10%) | 0/15 (0%) |
| No T2 hyperintensity | | 3/3 (100%) |  |

^*^Spinal levels with abnormal cord T2 weighted signal, assessed in sagittal plane. For first 3 rows listed, denominator is number of patients with the specific level imaged. For the remaining 6 rows in this section, denominator is total number of patients.

†Discrete lesions were those separated by ≥2mm of normal-appearing spinal cord signal on sagittal images ^‡^ T2 weighted signal abnormality. Excludes patients with only conus involved ^§^At location of area of maximal cross-sectional involvement. Excludes 1 case from period 1 with indeterminate involvement. ^‖^2 remaining cases from period 1 with anterior horn cell and some white matter involvement ^¶^Period 1: 3=ventral root at cervical or thoracic level; 1 indeterminate. Period 2: 1=ventral root at cervical level; 2=cauda equina.
