# Supplementary material for: A ten-year retrospective evaluation of acute flaccid myelitis at 5 pediatric centers in the United States, 2005–2014
Source: PLoS One. 2020 Feb 13;15(2):e0228671. doi: 10.1371/journal.pone.0228671 (PMC7018000; doi:10.1371/journal.pone.0228671)
Supplement: S1 Appendix — (DOCX) [file pone.0228671.s010.docx]

**Materials and methods**

**Pilot study**

The overall goal was to first identify the children that may have been a possible AFM case based on the hospitals’ issued spinal cord MRI report, and then review the spinal cord MR images, brain MR images, and medical charts of that subset to classify each of them as an AFM case or not. Study neuroradiologists/neurologists determined *a priori* the criteria necessitating review of the MR images: the report documented any of 4 key findings--myelitis, increased T2 signal in the spinal cord, cord edema, or cord infarction, without clear documentation of a known explanatory condition (e.g., tumor, established multiple sclerosis). Because the total number of spinal MRI reports issued per institution among children aged ≤18 years during the 10-year period were more than could be accurately reviewed in a timely manner (e.g., ~25,000 at CHOP), different strategies were explored by study staff at CHOP for their effectiveness in limiting the number of reports needing to be read to identify those with a key finding, but without missing possible AFM cases.

First, the “gold standard subset” (children whose MRI report contained a key finding) was identified for 2 randomly selected years (2008 and 2013), by staff reading each individual spinal cord MRI report that had been issued (n=2,723 reports). For 2013 and 2008, respectively, the gold standard subset consisted 11 and 14 children, respectively. Strategies to identify the gold standard subset were explored using data from 2013, and then the final strategy was tested against the data from 2008.

As the first strategy, we requested a list of all spinal cord MRIs performed at CHOP in patients that had a medical record diagnosis of myelitis, encephalomyelitis (including acute disseminated encephalomyelitis)*,* meningitis, encephalitis or multiple sclerosis, by searching inpatient and outpatient billing records by ICD-9 code (codes 323.0-323.99, 341.2-341.22, 323, 0.47.8, 047.9, 320.7, 320.0-322.90, 340; 366 patients over the 10-year period), but found this missed some of the gold standard subset **(Table A)**. A supplemental strategy was then explored based on literature for acute flaccid paralysis (AFP), using the list of ICD-9 codes published for ascertainment of AFP cases by Zangwill et al **(Table B).** A search of the hospital billing database using only the Zangwill ICD-9 codes, using the Zangwill ICD-9 codes to search the codes listed as the indication for performing the MRI, and searching the text of all MRI reports for the Zangwill text definition of the codes (e.g. other paralytic syndromes, quadriplegia, for ICD-9 344) each missed multiple children in the gold standard subset. However, when we modified this latter search using more common terms that would likely be included in MRI reports (Table B), the yield was much improved, and no children in the gold standard subset were missed for 2013. When this two-pronged search strategy was tested for the 2008 patients (1. Obtaining all the spinal cord MRI reports for any child who had had one of the listed discharge diagnosis ICD-9 diagnosis codes in the billing database, *and* 2. Searching all the spinal cord MRI reports to identify those that contained ≥1 of the 36 modified Zangwill text terms), we found that the children it had captured included 12 of the 14 in the 2008 gold standard subset, with only 2 missed (**Figure A)**. Neither of the 2 children in the gold standard subset that were missed by the search strategy was an AFM case based on review of the MR images and the medical chart: one child’s images demonstrated only a non-specific T2W abnormality that was not clinically significant, and the other child had cord infarction secondary to acute trauma.

**Other**

If a spinal MRI report was available that would have led to image review, but images were not able to be found or were unreadable, the MRI report and information from the chart review were used to make final AFM assessment. We did not attempt to obtain or review later follow-up clinical information or later scans of possible cases. Because we intentionally did not collect personal identifying information (PII), we did not attempt to determine if all 2014 cases had been reported by state health departments to CDC.

**Results**

At 4 sites combined (excluding STL) for the 10-year period, a total of 204 patients were identified that met criteria to have their spinal MR images reviewed by study radiologists; of the 202 that had images available and were read, 45 (22%) were deemed to be consistent with AFM, 30 (15%) in indeterminate category, and 127 (63%) not consistent with AFM. For 2 patients (one in 2009 and one in 2012), images were not available to be read by study neuroradiologists; based on hospital MRI reports and chart review, both of these patients were assessed to not be AFM. During 2005−2013, the average annual number of possible cases that had spinal MR images reviewed and were assessed as not consistent with AFM was 13 (range 9−20) and in 2014 there were 13 such patients.

At STL, where images from additional patients were reviewed (total 182), 3 (2%) were deemed to be consistent with AFM, 13 (7%) in indeterminate category, and 166 (91%) not consistent with AFM. At this site during 2005-2013, the average annual number of possible cases with MR images reviewed and assessed as not consistent with AFM was 16 (range 9-26) and in 2014 there were 18 such patients.

By comparing gender, age, and illness onset dates, we confirmed that no individual AFM or indeterminate case was counted by >1 site in a similar geographic area (i.e., CMKC and STL; CHOP and Hasbro).

**Table A**: Yield of search strategies in identifying potential cases of acute flaccid myelitis in pilot study

| Year | Total number of spinal cord MRI reports issued at CHOP, all individually read to find gold standard subset | **Number of patients in gold standard subset** (=children whose spinal cord MRI report contained a “key finding”, necessitating MR image review by study staff) | Search Strategy 1. Number of patients in gold standard subset identified via **ICD-9 discharge diagnosis code search** of billing database | Search Strategy 2. Number of patients in gold standard subset identified via **36-term text search of MRI report*** | Number of patients in gold standard subset missed via 2-pronged search strategy |
| --- | --- | --- | --- | --- | --- |
| 2013 | 1412 | 11 | 7 | 11 | 0 |
| 2008 | 1311 | 14 | 10 | 11 | 2 (1 with incidental non-specific T2 abnormality; 1 with infarction after trauma)  No missed cases consistent with myelitis or AFM |

*total number of spinal cord MRI reports that contained one of the 36-terms: 142 in 2013; 125 in 2008.

**Table B**: Text or ICD-9 codes used in some search strategies*

| **Zangwill diagnosis list** | **Zangwill ICD-9 code list** | **Modified text terms ultimately used in our Search Strategy 2** (search of spinal cord MRI report text) |
| --- | --- | --- |
| Botulism | 5.1 | Botulism |
| Acute poliomyelitis | 45 | Poliomyelitis |
| Meningitis and other diseases of CNS due to enterovirus; other nonarthropod-borne viral disease of the central nervous system | 047, 048, 049 | Meningitis |
| Meningitis due to virus, not otherwise specified | 321.2 | Meningitis |
| Encephalitis following immunization, encephalitis, unspecified; transverse myelitis | 323.5, 323.9 | encephalitis, encephalomyelitis, myelitis |
| Anterior horn cell disease; disease of spinal cord | 335.29, 335.8, 335.9 | anterior horn, gray matter |
| Multiple sclerosis; demyelinating diseases of the central nervous system | 340, 341 | multiple sclerosis, ADEM, demyelinating, demyelination |
| Hemiplegia and hemiparesis; infantile hemiplegia, infantile cerebral palsy | 343, 343.4, 343.8 | hemiplegia, hemiparesis, cerebral palsy |
| Other paralytic syndromes, quadriplegia | 344 | paralysis, quadriplegia |
| Unspecified disorders of the nervous system | 349.9 | *None – eliminated from search* |
| Cranial nerve, nerve root, and plexus disorders | 350, 351, 352, 353 | neuropathy, neuritis, nerve root |
| Mononeuritis | 354,355 | Mononeuritis |
| Peripheral Neuropathy | 356 | Neuropathy |
| Acute infective polyneuritis, including Guillain-Barre Syndrome | 357 | polyradiculoneuropathy, Guillain-Barre |
| Muscular dystrophies, channelopathies, and other myopathies | 359 | Muscular dystrophy, myopathy |
| Acute cerebrovascular disease | 436 | stroke, infarction, vasculitis |
| Discitis, brachial neuritis, radiculitis | 722.9, 723.4,724.4, 729.2 | discitis, neuritis, radiculitis |
| Infective myositis | 728 | Myositis |
| Transient limb paralysis or monoplegia | 781.4 | monoplegia, paralysis |
| Trauma of spinal cord, nerve plexuses, peripheral nerves | 952-957 | cord trauma, nerve trauma |
| Tick paralysis | 989.5 | Paralysis |
| Acute flaccid paralysis | 359.9 | Paralysis |

*Modified from Zangwill KM, Yeh SH, Wong EJ, et al. Paralytic syndromes in children: epidemiology and relationship to vaccination. Pediatr Neurol. 2010 Mar; 42(3):206-12

**Figure A. Pilot study schematic**

**
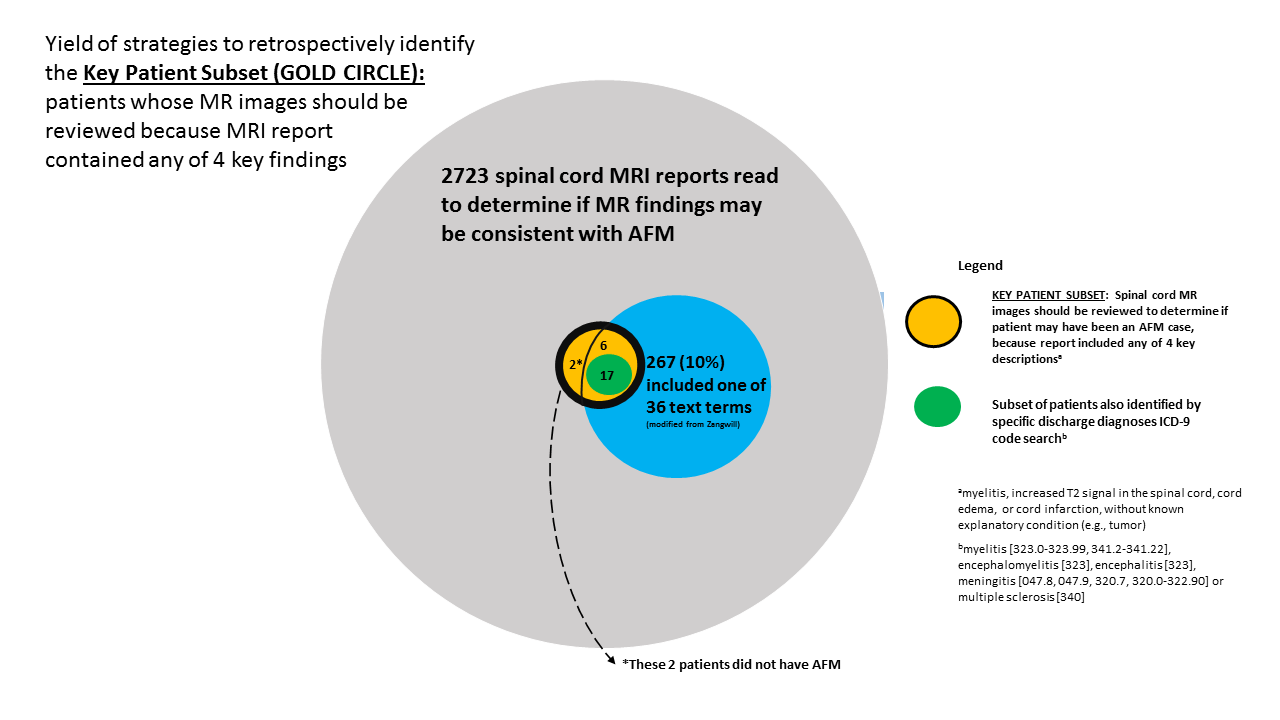
**
